# Prognostic Value of Cancer Stem Cell Marker Aldehyde Dehydrogenase in Ovarian Cancer: A Meta-Analysis

**DOI:** 10.1371/journal.pone.0081050

**Published:** 2013-11-25

**Authors:** Shuyan Liu, Chengfei Liu, Xiaoyun Min, Yuanyuan Ji, Na Wang, Dan Liu, Jiangyi Cai, Ke Li

**Affiliations:** 1 Core Research Laboratory, The Second Affiliated Hospital, School of Medicine, Xi’an Jiaotong University, Xi’an, China; 2 Department of Reproductive Medicine, The First Affiliated Hospital, School of Medicine, Xi’an Jiaotong University, Xi’an, China; 3 Department of Pathology, Aerospace General Hosipital, Xi’an, China; National Cancer Center, Japan

## Abstract

**Objective:**

Aldehyde dehydrogenase (ALDH) has recently been reported as a marker of cancer stem-like cells in ovarian cancer. However, the prognostic role of ALDH in ovarian cancer still remains controversial. In this study, we aimed to evaluate the association between the expression of ALDH and the outcome of ovarian cancer patients by performing a meta-analysis.

**Methods:**

We systematically searched for studies investigating the relationships between ALDH expression and outcome of ovarian cancer patients. Only articles in which ALDH expression was detected by immunohistochemical staining were included. A meta-analysis was performed to generate combined hazard ratios (HRs) with 95% confidence intervals (CIs) for overall survival (OS) and disease-free survival (DFS).

**Results:**

A total of 1,258 patients from 7 studies (6 articles) were included in the analysis. Our results showed that high ALDH expression in patients with ovarian cancer was associated with poor prognosis in terms of Os (HR, 1.25; 95% CI, 1.07-1.47; P = 0.005) and DFS (HR, 1.58; 95% CI, 1.00-2.49; P = 0.052), though the difference for DFS was not statistically significant. In addition, there was no evidence of publication bias as suggested by Begg’s and Egger’s tests (Begg’s test, P = 0.707; Egger’s test, P = 0.355).

**Conclusion:**

The present meta-analysis indicated that elevated ALDH expression was associated with poor prognosis in patients with ovarian cancer.

## Introduction

Ovarian cancer is the most lethal of all gynecological malignancies and the seventh leading cause of cancer death among women worldwide [1]. Over 90% of ovarian cancers arise from the epithelial surface of the ovary, the rest from germ cells or stromal cells. The epithelial ovarian cancers are classified as serous (30–70%), endometrioid (10–20%), mucinous (5–20%), clear cell (3–10%), and undifferentiated (1%) [2]. It is disproportionally deadly due to the absence of either specific symptoms or effective screening and early detection strategies, leading to over 70% of patients being diagnosed with advanced stage disease, in which the 5-year survival rate is only 30% [3]. Hence, it is necessary to identify prognostic factors to predict the outcomes of patients, which could be effective in making strategies and improving survival for ovarian cancer. Age, performance status, tumor histology and residual tumor volume are considered as independent predictors of prognosis in ovarian cancer patients with advanced stage [4]. However, these factors are insufficient to predict the outcomes for the individual patient. Identifying molecular biological prognostic factors could enable to predict patients' outcomes more accurately and provide novel therapeutic targets.

The cancer stem cell model suggests that in many cancers, tumor initiation and propagation is driven by a population of self-renewing tumor cells known as cancer stem cells (CSCs) [5]. Accumulating evidence has proposed that CSCs are responsible for tumor progression, relapse, metastases, and therapeutic resistance, thus indicating poor prognosis [6,7]. Therefore, the identification of CSCs has become an important issue particularly in the context of potential therapeutic targeting. Aldehyde dehydrogenase (ALDH) has been one of the most frequently used biomarkers in CSC-related research, with a career that started with the isolation of ALDH+ CSCs from breast cancer [8]. Since then, the isolation of putative CSCs by ALDH activity has been reported from a wide range of solid tumors, including those of colon, bladder, prostate, lung, pancreas, head and neck, endometrium and melanoma [9-19].

Landen et al. were the first to isolate putative CSCs in ovarian cancer by high ALDH activity and showed that high ALDH expression predicts poor outcome of ovarian cancer patients [20]. Consistence with that, other studies also found that ALDH was a predictor of poor prognosis in ovarian cancer [21-24]. However, Chang et al. showed that ALDH expression correlates with favorable prognosis in ovarian cancer [25]. Moreover, Ricci et al. showed that no correlation was found between the expression of ALDH and survival of ovarian cancer [26]. Insufficient samples and some other factors have resulted in controversial results of different clinical studies. The present meta-analysis aims to determine the value of ALDH as a prognostic marker for ovarian cancer.

## Methods

### Literature search strategy

We comprehensively searched PubMed, Cochrane library, EMBASE, Web of Science and CBM electronic databases for relevant articles published until August 1st, 2013. Search terms included terms for Ovarian Cancer (“Ovarian Neoplasm”, “Ovarian Carcinoma”, “Ovarian Cancer”, “Ovarian Tumor”) and Aldehyde Dehydrogenase ( “Aldehyde Dehydrogenase”, “ALDH”). The reference lists of relative articles were also screened manually to further identify potential studies.

### Criteria for inclusion and exclusion

The following inclusion criteria were used in order to ensure the high quality of this article: (1) patients with distinctive Ovarian Cancer diagnosis by pathology; (2) full length paper with sufficient data on survival and ALDH expression; (3) ALDH expression was measured by immunohistochemistry (IHC) method. The following studies were excluded: (1) articles about cell lines or animals; (2) review articles without original data; (3) studies lacking information on survival.

### Data extraction and quality assessment

The following information were retrieved independently by 2 authors (SY Liu and CF Liu) from the final set of literatures: publication year, first author, number of patients enrolled, histology and disease stage, cutoff value, Hazard ratio (HR) and 95% confidence interval (CI) as well as the other related events. Discrepancies were resolved by discussion and consensus. If the above information were not mentioned in the original study, the item was treated as “Not Available (NA)”. 

Quality assessment was conducted in each of the available studies by using the Newcastle-Ottawa quality assessment scale for cohort studies. This scale allows for assessment of patient population and selection, study comparability, follow-up, and outcome of interest. Interpretation of the scale is performed by awarding points, or ‘‘stars,’’ for high-quality elements. Stars are then added up and used to compare study quality in a quantitative manner.

### Statistical analysis

HR and 95% CI were used as the effective value to measure the impact of ALDH expression on survival of ovarian cancer patients in this meta-analysis. In the individual study, some of them provided HR and 95% CI directly. For some other studies not given these data clearly, we calculated from available data or Kaplan-Meier survive cure by using Engauge Digitizer version 4.1 (free software down-loaded from http://sourceforge.net). If a study provided both the results of multivariate analysis and univariate analysis, we chose the former. Heterogeneity was assessed by the Chi-squared test. The I^2^ value was used to evaluate the heterogeneity (I^2^ = 0-50%, no or moderate heterogeneity; I^2^ > 50%, significant heterogeneity) [27]. Begg's test and Egger's test were performed to identify the possibility of publication bias [28,29]. Fixed-effect model was used if there was no significant heterogeneity. Otherwise, the random-effect model was used. Sensitivity analysis was performed to examine the stability of the pooled results. By convention, an observed HR > 1 implied a poor survival for the group with increased ALDH expression. The impact of increased ALDH expression on survival was considered statistically significant if the 95% CI did not overlap with 1. All the p-values were two sided, and p < 0.05 was considered statistically significant. All statistical analyses were conducted with STATA 12.0.

## Results

### Study selection and characteristics

**Figure 1:**
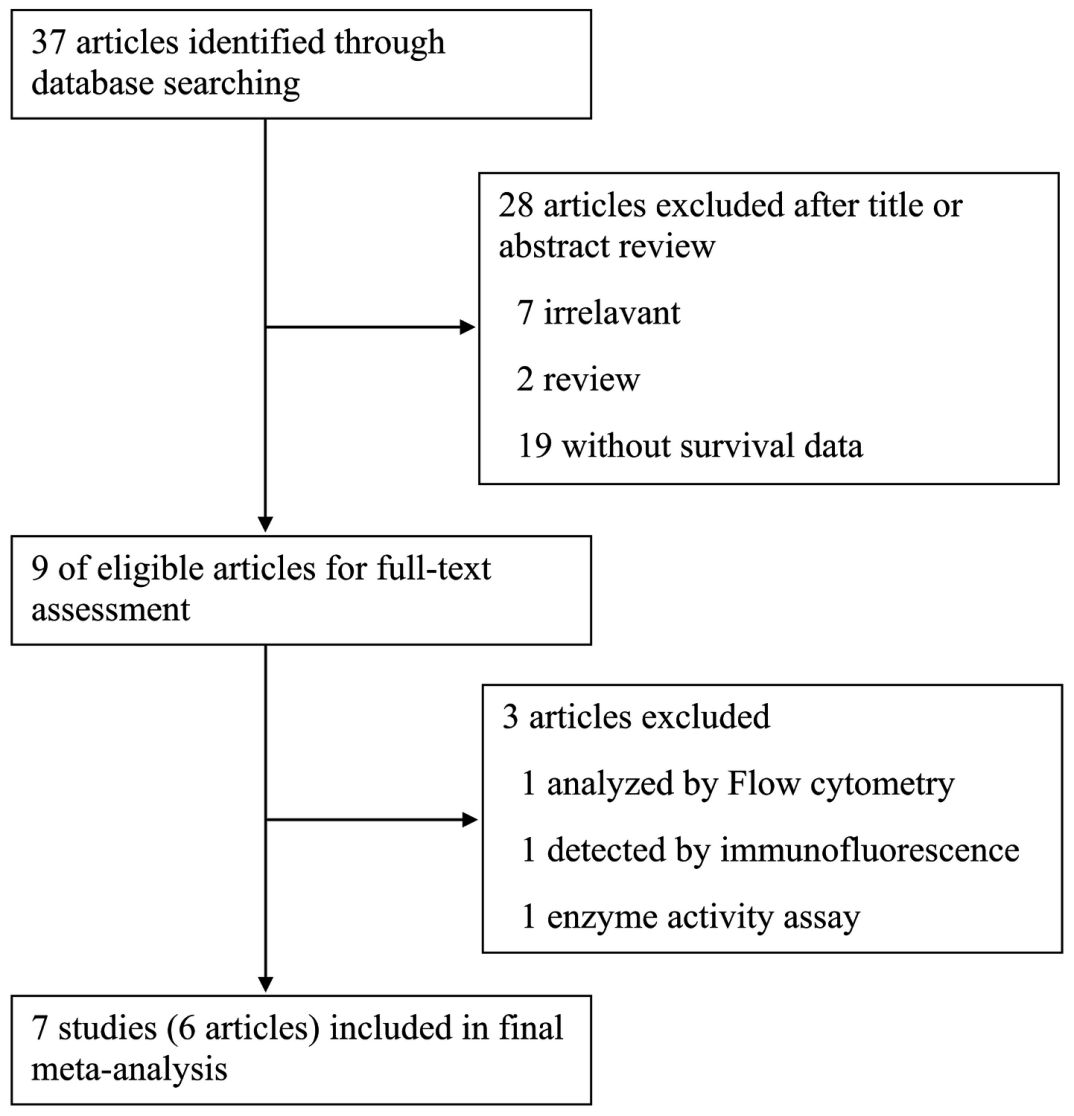
Flow diagram showing inclusion and exclusion of studies.

As shown in Figure 1, a total of 37 articles were identified initially using the search strategy above. Titles and abstracts of all identified studies were reviewed to exclude those that were clearly irrelevant. A total of 9 potentially relevant articles were fully reviewed with the full text. Among them, 3 articles were excluded because of the detection method for ALDH expression: 1 was by flow cytometry analysis, 1 by immunofluorescence and 1 by enzyme activity assay. Finally, 7 studies (6 articles) were selected for the present meta-analysis [20-25].

All the 7 studies were performed for detection of ALDH isoform 1A1 expression in the cytoplasm of tumor cells in ovarian cancer tissues by IHC analysis. Other main characteristics of the included studies were shown in Table 1. The studies were conducted in 4 countries (USA, Taiwan, Germany and Japan) and published between 2009 and 2013. A total of 1,258 patients with a median of 84 (ranged from 37 to 440) were included. The reported median age of patients ranged from 21 to 89 years across the eligible studies. The follow-up period ranged from 4 to 475 months. 1 study defined the cut off value by complex score combining intensity and percentage of ALDH expression, while other studies only used the percentage of ALDH expression to define positive expression with the cut off value varied from 1% to 50%. There were 6 studies utilized both overall survival (OS) and disease-free survival (DFS) to assess the prognostic value of ALDH expression in ovarian cancer patients and 1 study used only OS as the indicator. Among all of the included studies, HR and 95%CI were obtained from the original articles directly in 2 studies. For the remaining 5 studies, HR and 95% CI were extrapolated from Kaplan-Meier curves. Out of the 7 studies, 3 studies provided the results of multivariate analysis and 3 studies provided HRs from univariate analysis and another one did not provide information of statistical method.

**Table 1:** Characteristics of the included studies.

**First author**	**Year**	**Country**	**No. of Patients**	**Age (y)**	**Follow-up (month)**	**Histologic type**	**FIGO disease stage**	**Study quality^*#*^**	**Cutoff scores**	**ALDH high/low**	**Survival analysis**	**HR (95% CI)**	**Analysis**
Chang	2009	USA	440	60 (21–89)	Median 96	S 266, E35, M5, C14, T6, O 116	I 32, II 30, III 305, IV 72, unknown 3	8	>20%	87/353	OS	0.92(0.67-1.27) *	Multivariate
											DFS	0.79(0.61-1.02) *	
Deng	2010	USA	439	NA	101 (4-475)	S 439	NA	6	≥10%	175/264	OS	1.27(1.03-1.56) *	NA
											DFS	1.77(1.37-2.29) *	
Landen	2010	USA	65	62.2 (34-89)	>100	S 65	III 48, IV 17	7	>1%	48/17	OS	1.39(0.69-2.80) *	Univariate
											DFS	2.03(1.16-3.57) *	
Wang	2012	Taiwan	84	≤50% (52.0±1.9)	60	S 61, M 14, E 3, C 6	I –II 27, III–IV 57	6	>50%	28/56	OS	2.43(1.12-5.28)	Multivariate
				>50% (57.0±2.8)							DFS	1.70(0.77-3.77)	
Liebscher	2013	Germany	131	59.6 (34-87.1)	>100	S 131	I 9, II 6, III 105, IV 11	7	IRS≥4	42/89	OS	2.01(1.03-3.93)	Multivariate
Kuroda	2013	Japan	62	56.6±11.4	60	S 62	I –II 10, III–IV 52	7	>20%	34/28	OS	1.63(0.34-7.89) *	Univariate
											DFS	1.95(0.95-4.00) *	
Kuroda	2013	Japan	37	52.2±9.4	60	C 37	I –II 23, III–IV 14	7	>15%	18/19	OS	3.62(0.47-27.88) *	Univariate
											DFS	3.97(0.60-26.19) *	

FIGO, International Federation of Gynecology and Obstetrics; IRS, immunoreactivity score; OS, overall survival; DFS, disease-free survival; NA, not available; HR, hazard ratio; CI, confidence interval; S, serous; M, mucinous; E, endometrioid; C, clear cell; T, transitional cell; O, others.

^#^ Study quality was judged based on the Newcastle-Ottawa Scale (range, 1-9 stars).

* estimated.

### ALDH expression and prognosis of ovarian cancer

**Figure 2:**
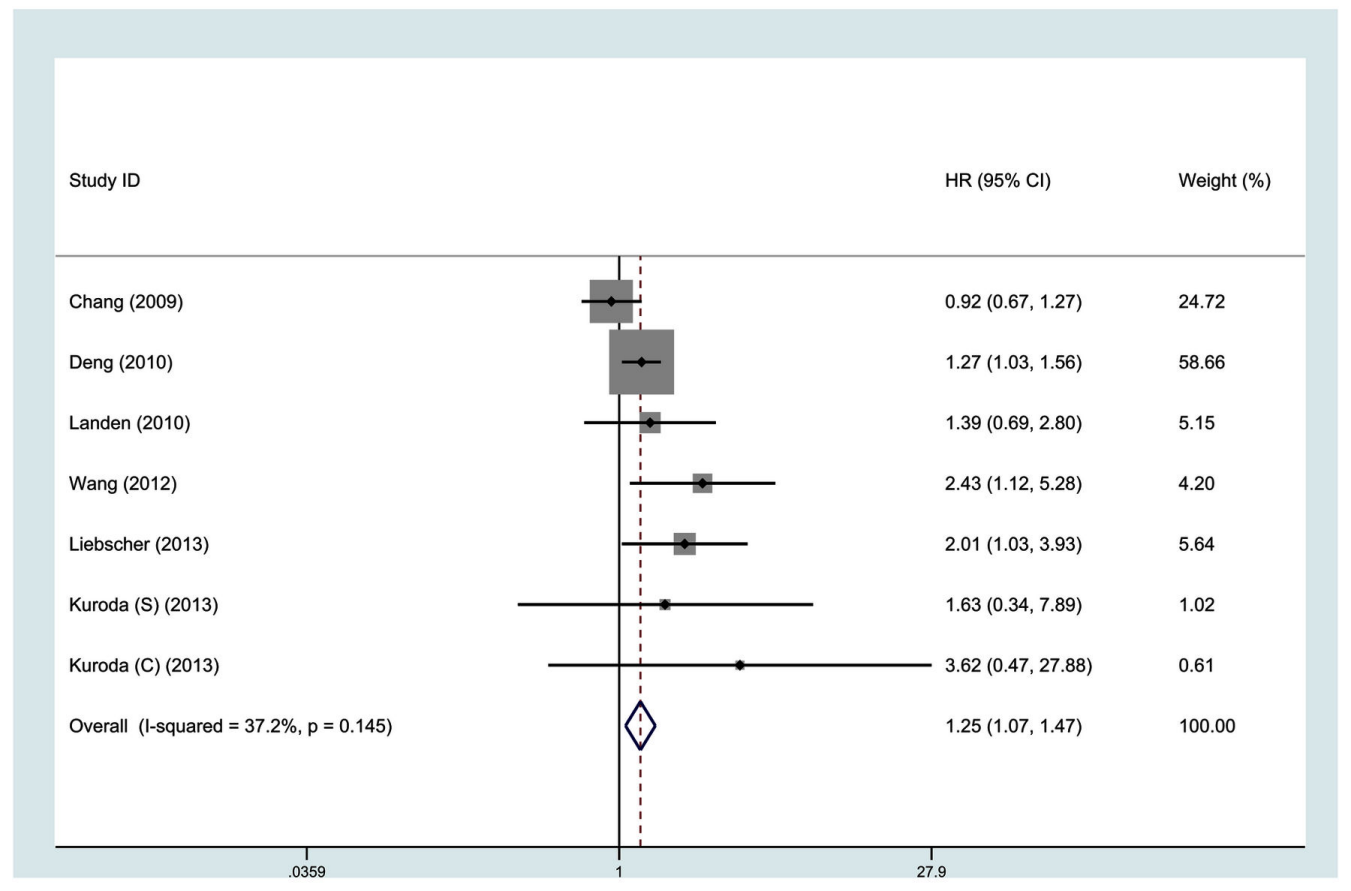
Meta-analysis of 7 evaluable studies assessing OS according to methods of analysis by a fixed-effects model.

**Figure 3:**
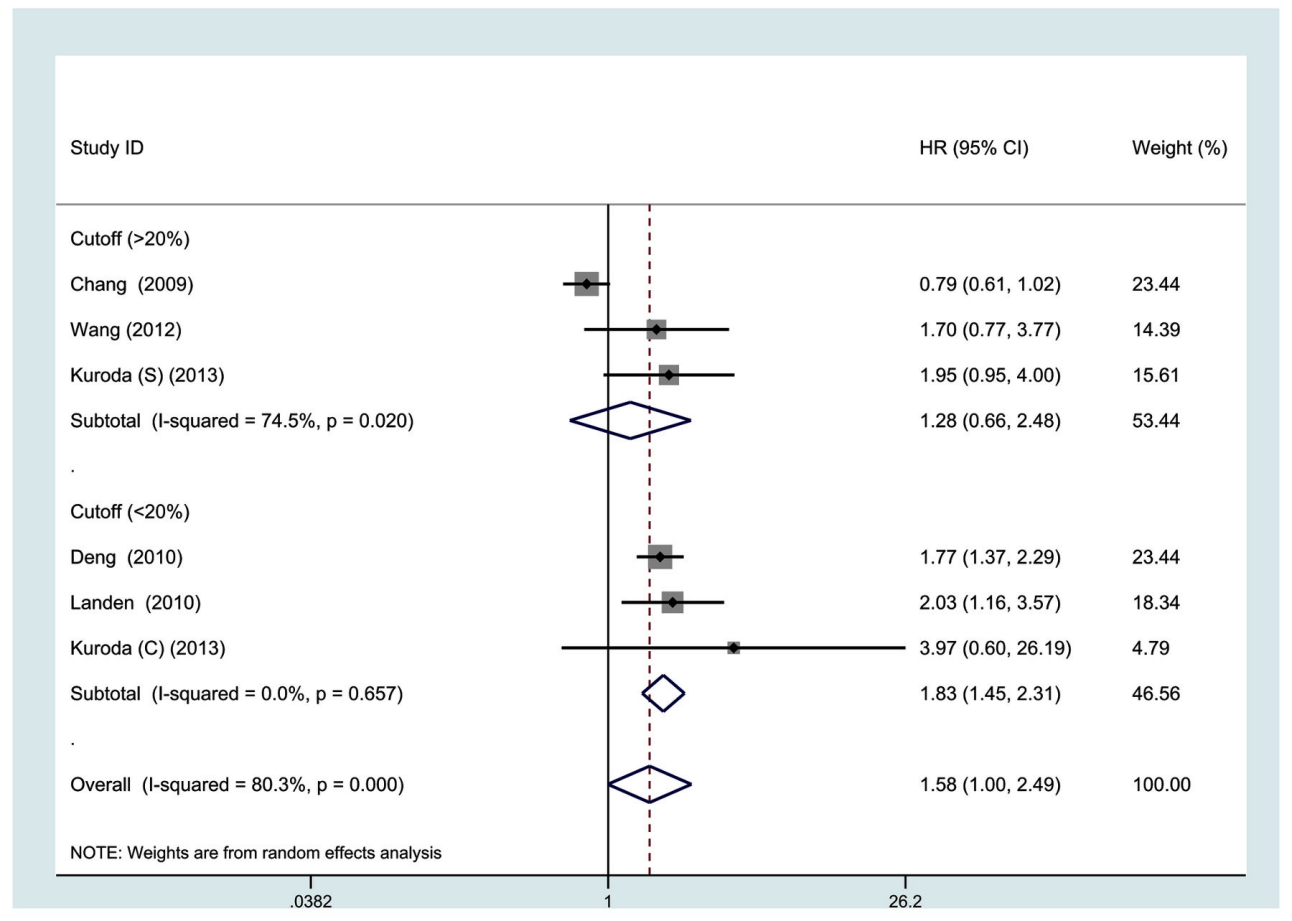
Meta-analysis of 6 evaluable studies assessing DFS according to methods of analysis by a random-effects model.

All 7 studies investigating OS were pooled into the meta-analysis. As shown in Figure 2, high ALDH expression correlates with poor OS (HR, 1.25; 95% CI, 1.07-1.47). No significant heterogeneity was observed among the studies (I^2^ = 37.2%, p = 0.145). Data on DFS were available from 6 studies. As shown in Figure 3, high ALDH expression was not significantly associated with poor DFS (HR, 1.58, 95% CI, 1.00-2.49, I^2^ = 80.3%).

Table 2 shows the results of the subgroup meta-analyses. The subgroup of studies from univariate analysis showed that high ALDH expression predicts poor DFS (HR, 2.07; 95% CI, 1.35-3.19), but not OS (HR, 1.55; 95% CI, 0.84-2.86); while the subgroup of studies from multivariate analysis showed no association of ALDH expression with OS or DFS. When grouped according to study region of individual studies, the combined HRs of Asian studies in OS and DFS were 2.36 (1.22-4.56) and 1.94 (1.16-3.24), respectively, indicating ALDH is an indicator of poor prognosis of OS and DFS in Asian patients. The subgroup meta-analysis of studies with the cutoff score < 20% showed high ALDH expression was associated with poor OS (HR, 1.29; 95% CI, 1.06-1.57; I^2^=0%) and DFS (HR, 1.83; 95% CI, 1.45-2.31; I^2^=0%) of ovarian cancer patients. When grouped according to follow-up period, both subgroups showed that ALDH expression was a predictor for poor OS (HR, 1.21; 95% CI, 1.02-1.42 and HR, 2.36; 95% CI, 1.22-4.56). Subgroup meta-analysis of studies with follow-up period of less than 60 months showed that high ALDH expression was also a predictor for poor DFS (HR, 1.94; 95% CI, 1.16-3.24) in ovarian cancer. When restricted to studies about the specific histological type of serous carcinoma, high ALDH expression was significantly associated with poor OS (HR, 1.33; 95% CI, 1.10-1.61; I^2^=0%) and DFS (HR, 1.83; 95% CI, 1.46-2.28; I^2^=0%). Among studies with median age of < 60 y, we also observed a statistically significant effect of ALDH expression on OS (HR, 2.18; 95% CI, 1.36-3.49; I^2^=0%) and DFS (HR, 1.94; 95% CI, 1.16-3.24; I^2^=0%) in ovarian cancer patients.

**Table 2:** Associations between ALDH expression and ovarian cancer prognosis grouped by selected factors.

**Variables**	**No. of studies**	**No. of patients**	**HR (95% CI)**	**I^2^ (%)**
***OS***	7	1258	1.25 (1.07-1.47)	37.2
Analysis				
Multivariate	3	655	1.18 (0.90-1.54)	75.3
Univariate	3	164	1.55 (0.84-2.86)	0.0
Ethnicity				
Asian	3	183	2.36 (1.22-4.56)	0.0
Non-Asian	4	1075	1.23 (0.94-1.60)	44.3
Cutoff				
>20%	3	586	1.40 (0.67-2.92)	63.2
<20%	3	541	1.29 (1.06-1.57)	0.0
Follow-up (m)				
>60	4	1075	1.21 (1.02-1.42)	44.3
≤60	3	183	2.36 (1.22-4.56)	0.0
Histological type				
S	4	697	1.33 (1.10-1.61)	0.0
C	1	37	3.62 (0.47-27.88)	0.0
M	2	524	1.40 (0.54-3.59)	80.6
Age ( y)				
≥60	2	505	1.00 (0.72-1.39)	9.4
<60	4	314	2.18 (1.36-3.49)	0.0
***DFS***	6	1157	1.58 (1.00-2.49)	80.3
Analysis				
Multivariate	2	524	1.05 (0.51-2.18)	69.1
Univariate	3	164	2.07 (1.35-3.19)	0.0
Ethnicity				
Asian	3	213	1.94 (1.16-3.24)	0.0
Non-Asian	3	944	1.38 (0.74-2.59)	91.0
Cutoff				
>20%	3	586	1.28 (0.66-2.48)	74.5
<20%	3	541	1.83 (1.45-2.31)	0.0
Follow-up (m)				
>60	3	944	1.38 (0.74-2.59)	91.0
≤60	3	183	1.94 (1.16-3.24)	0.0
Histological type				
S	3	566	1.83 (1.46-2.28)	0.0
C	1	37	3.97 (0.60-26.19)	0.0
M	2	524	1.05 (0.51-2.18)	69.1
Age ( y)				
≥60	2	505	1.22 (0.49-3.08)	88.8
<60	3	183	1.94 (1.16-3.24)	0.0

OS, overall survival; DFS, disease-free survival; HR, hazard ratios; CI, confidence intervals; S, serous; C, clear cell; M, mixed.

### Publication bias analysis

**Figure 4:**
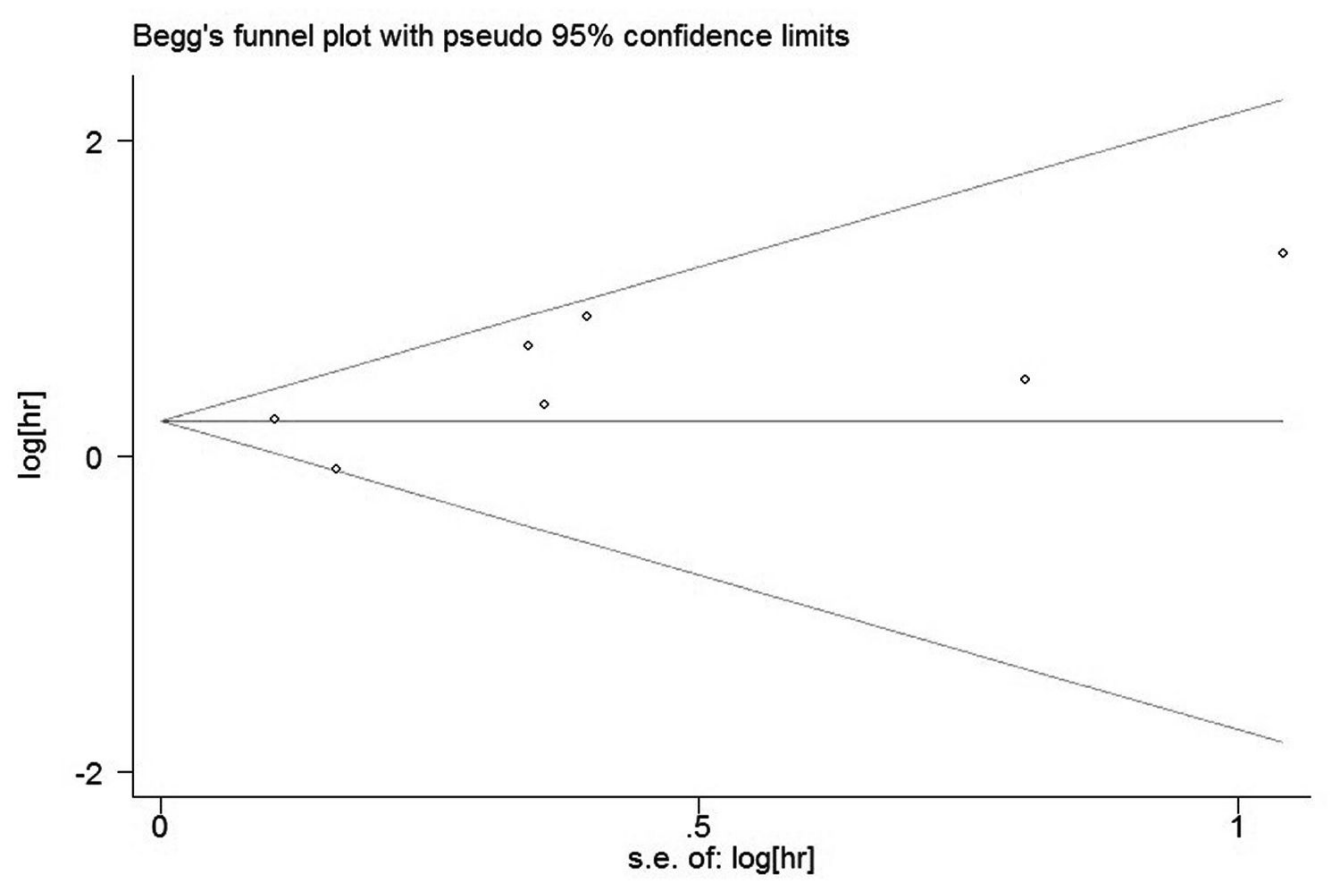
Funnel plot of the meta-analysis assessing ALDH Expression and OS in ovarian cancer.

**Figure 5:**
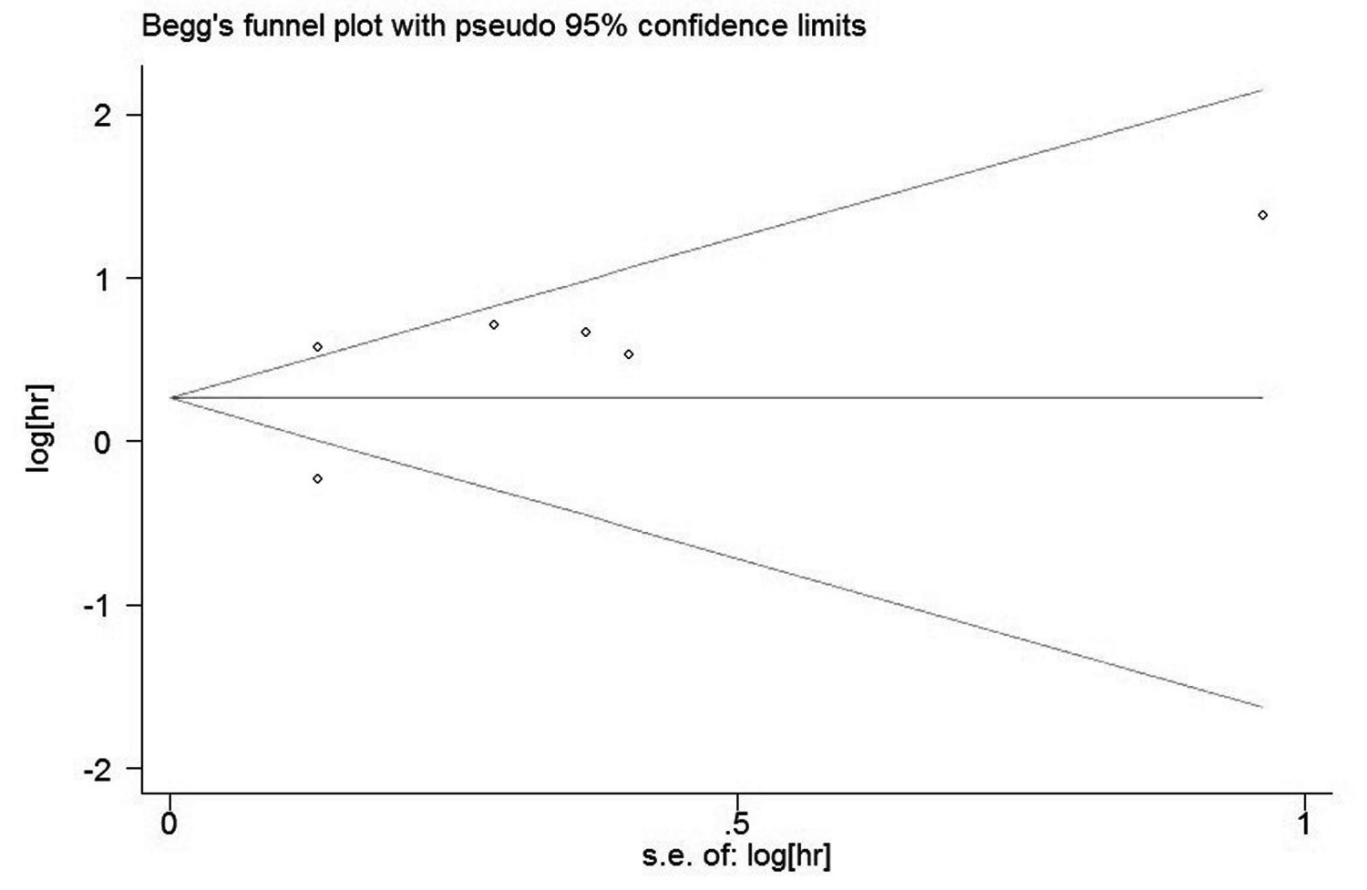
Funnel plot of the meta-analysis assessing ALDH Expression and DFS in ovarian cancer.

The funnel plots presented no proof of obvious publication bias for studies in either of the two outcomes (Figure 4 and Figure 5). There was no evidence for significant publication bias in OS (Begg’s test, P = 0.707; Egger’s test, P = 0.355) and DFS (Begg’s test, P = 0.230; Egger’s test, P = 0.162) studies.

### Sensitivity analysis

**Figure 6:**
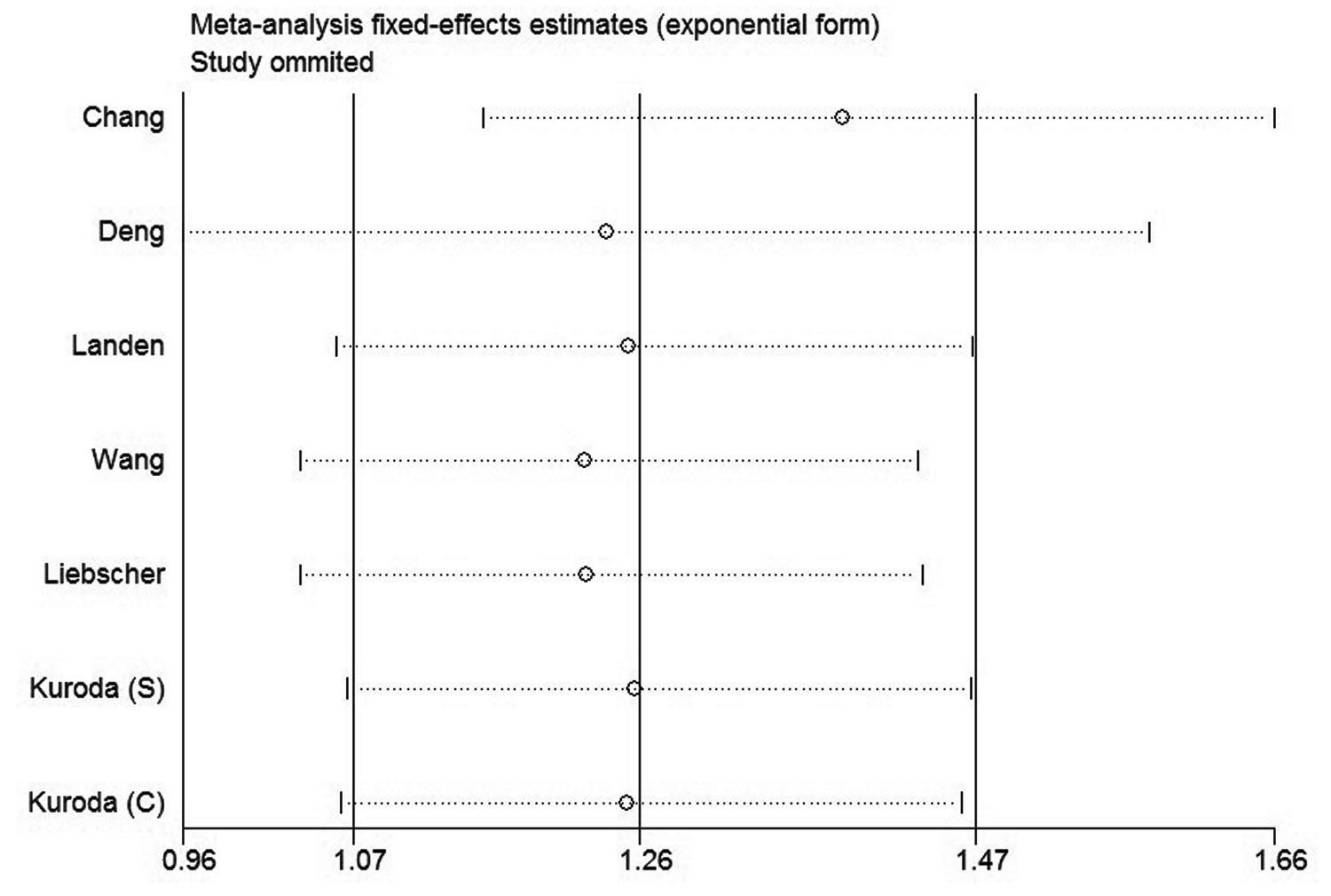
Sensitivity analysis of all the studies assessing OS.

**Figure 7:**
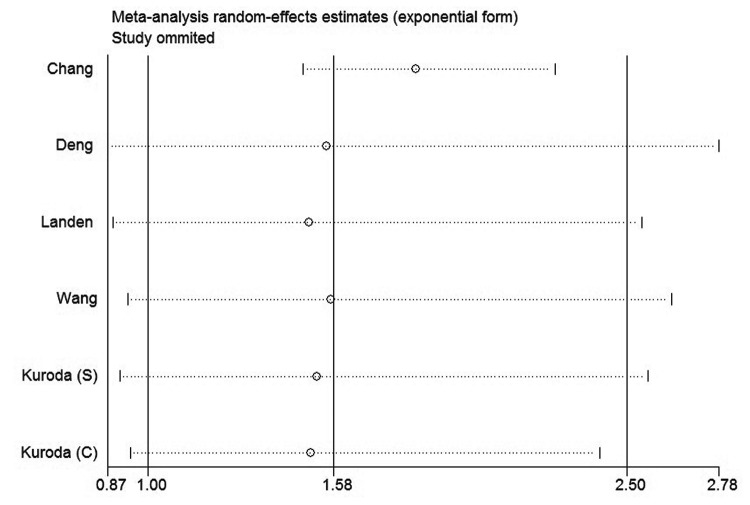
Sensitivity analysis of all the studies assessing DFS.

In order to gauge results stability, a sensitivity analysis, in which one study was deleted at a time, was performed. The results were shown in Figure 6 and Figure 7. Both of the corresponding pooled HRs of OS and DFS were not significantly changed, suggesting the robustness of our results.

## Discussion

According to the CSC hypothesis, only a small fraction of cells, namely the CSCs, within a tumor is multipotent and has the capability of indefinite proliferative potential that drive the formation and growth of tumors [5]. The first evidence for the existence of CSCs came from acute myeloid leukemia, in which a rare subset comprising 0.01–1% of the total population could induce leukemia when transplanted into immunodeficient mice [30]. This concept was then extended to solid tumors. The first solid malignancy from which CSCs were identified and isolated was breast cancer. Al-Hajj et al. described a CD44^+^CD24^-/low^ cell population that was significantly enriched for tumor-initiating capacity [31]. There is now increasing evidence for CSCs in a variety of solid tumors. This new paradigm has remarkable implications for cancer therapy because it suggests that our current therapies are more successful at eradicating non-CSCs than CSCs [32,33]. Consequently, the purification and characterization of CSCs could lead to the identification of better targets for therapeutic intervention. 

A number of cell surface and function markers have proved useful for the isolation of subsets enriched for CSCs, including CD133, CD44, CD24, CD90, epithelial cell adhesion molecule (EpCAM), and ATP-binding cassette B5 (ABCB5), cytosolic detoxifying enzyme ALDH as well as Hoechst 33342 exclusion by the side population cells [7]. Among these markers, ALDH was a widely used marker for isolating CSCs in a range of solid tumors, including ovarian cancer [8-14,19,20]. Moreover, high ALDH expression was reported to be associated with poor prognosis in breast cancer [8,34-36].

Although high expression of CSC markers are usually considered as a prognosis of poor outcome, several contradictions to this generalization exist in published studies on the putative CSC-marker ALDH. 5 studies in this meta-analysis concluded that high ALDH expression is a predictor for poor prognosis [20-24]. Ricci et al. showed no correlation between the expression of ALDH and survival of ovarian cancer [26]. However, Chang et al. found that high percentage of cells expressing ALDH was associated with a longer overall survival time and disease-free survival time [25]. In contrast to its function in breast cancer, ALDH was a favorable prognostic factor in ovarian cancer. They also found that high expression of ALDH was associated with early-stage disease. Furthermore, multivariate Cox proportional hazards regression analysis showed that the early stage of disease was strongly associated with longer overall survival and disease-free survival.

This meta-analysis showed that estimates of the significance of ALDH expression vary substantially between studies. However, we found that high ALDH expression was associated with poor OS and DFS in patients with ovarian cancer, although not significant for DFS. The results increase the likelihood that high ALDH expression is an independent risk factor for ovarian cancer.

To the best of our knowledge, this is the first meta-analysis to evaluate the prognostic role of ALDH expression in ovarian cancer. Sub-group analysis identified several important findings. A prominent association was observed between ALDH and poor OS and DFS when studies set the cutoff score at < 20%. It was reported that ALDH expression was limited to a small subpopulation of tumor cells. In breast cancer, ALDH-positive cells represented an average of 5% of cells in tumors expressing ALDH, which was consistent with the idea that cancer stem cells constitute a minority of the tumor population. Only two of the 481 tumors had ALDH staining in the vast majority of the cell population [8]. We speculate that when the cutoff was > 20%, a majority of cases with high ALDH expression were excluded and assigned to the group of low ALDH expression. Subgroup analysis by histological type showed that high ALDH expression was significantly associated with poor OS and DFS in serous ovarian carcinomas. In addition, subgroup analysis by study region revealed that higher ALDH expression was associated with poor OS and DFS in studies performed in Asia. This finding should be interpreted with caution owing to the small number of studies. Further studies are needed to evaluate whether the prognostic role of ALDH differs with patient ethnicity.

There are several limitations to the current meta-analysis. First, the number of studies included is relatively small. Second, heterogeneity was found in the main analysis. This may have arisen from the different characteristics of the subjects and the various histological types of ovarian cancer. Furthermore, the methodology for immunohistochemistry could affect heterogeneity due to the various detecting antibodies against ALDH and the application of different cutoff values for determining high ALDH levels. Third, we were unable to perform subgroup analysis by FIGO stage, grade, and histological type to evaluate the pooled HR for OS and DFS because diverse subjects were included in each study.

In conclusion, we found that high ALDH expression may be an independent risk factor for ovarian cancer prognosis. Based on the current findings, assessing ALDH expression could provide better prognostic information for patients with ovarian cancer and be used as a novel therapeutic target. Further large-scale cohort studies are needed to validate our results.

## Supporting Information

Checklist S1 **PRISMA checklist.**
(DOC)
